# The Forty-Seventh Annual Meeting of the Mississippi Valley Association of Dental Surgeons

**Published:** 1891-05

**Authors:** 


### PROCEEDINGS.

### The Forty-Seventh Annual Meeting of the Mississippi Valley Association of Dental Surgeons.

CINCINNATI, OHIO, March 10th, 11th and 12th.

The Association met in Lincoln Club Hall, corner Eighth and Race streets, Tuesday morning March 12th, 1891, with President, Dr. M. H. Fletcher, of Cincinnati, Ohio, in the Chair.

Prayer was offered by Dr. James Leslie, of Cincinnati, Ohio.

The minutes of the previous meeting were read and approved.

Dr. J. R. Callahan, Chairman of the Executive Committee, reported that the result of the work of the Committee was represented in the programme for the meeting, that the plan adopted at the last meeting, of appointing members of the Executive Committee from each of the large cities of the Mississippi Valley seemed to be a good one, and, that as a rule, each member had done good work for the meeting, and this was especially so in regard to Dr. M. Stout, the member from Chicago.

The Committee on membership was called and reported that up to the present time no names had been proposed for membership.

Dr. Frank Hunter, Treasurer, reported the balance on hand to be $118.34.

On motion of Dr. H. A. Smith, Dr. J. J. R. Patrick, of Belville, Ill., was to be given the privilege of the floor when he was ready to speak upon the subject of the Examination of Crania.

On motion of Dr. J. Taft, 10:30 on Wednesday was the hour set apart for the hearing of Dr. E. S. Talbot's paper, and two o'clock the same day, the hour for Dr. B. M. Rickett's paper.

The President appointed the following committees on Necrology: Dr's J. Taft, F. W. Sage, and H. T. Smith.

Upon motion, two o'clock in the afternoon was the hour set apart for hearing Dr. F. W. Sages's paper and the discussion of Dr. Fletcher's paper.

Dr. J. W. Jay, of Richmond, Ind., made some interesting remarks upon the subject of "Dentistry Thirty Years Ago and Now," followed by Dr. James Leslie, on the same subject.

On motion, the Association adjourned, to meet at two o'clock in the afternoon.

### AFTERNOON SESSION, TUESDAY, MARCH 10TH, 1891.

Association met, with Dr. M. H. Fletcher in the Chair.

On motion, the portion of Dr. Fletcher's paper, relating to treatment of teeth was re-read, and this was followed by Dr. F. W. Sage's paper "Phagosytosis in Dental Lesions." The two papers were then considered and discussed together by Dr.'s J. Taft, C. M. Wright, H. A. Smith, Frank Hunter, C. P. Gray, G. S. Junkerman. Dr. Dennis, Dr. J. R. Callahan, J. W. Jay and M. H. Fletcher.

On motion, the subject was passed, and the paper, "Why Copper Amalgam Sometimes Washes in the Mouth," was read by Dr. W. B. Ames, of Chicago, Ill.

On motion, the Association adjourned. The discussion of Dr. Ames' paper was made the first subject for Wednesday morning.

### SESSION OF WEDNESDAY MORNING, MARCH 11TH, 1891.

Association met, with President M. H. Fletcher in the Chair.

The discussion of Dr. Ames' paper was opened by Dr. L. E. Custer, of Dayton, Ohio, followed by Drs. J. S. Cassidy, E. G. Betty, C. M. Wright, J. Taft, James Leslie and H. A. Smith, who presented for examination some amalgam crowns, contours made by Dr. Arkovoy, of Budapest, Hungary.

On motion of Dr. J. Taft, Dr.'s J. J. R. Patrick, of Belleville, Ill., and E. S. Talbot, of Chicago, were unanimously elected honorary members of the Association.

Dr. E. S. Talbot, of Chicago, read a paper, "Scientific Investigation of the Jaws and Teeth." The discussion was opened by Dr. J. J. R. Patrick.

At the hour of the adjournment, it was moved that the Association adjourn and the discussion of Dr. Talbot's paper be made the first order for afternoon session, passed and Association adjourned.

AFTERNOON SESSION, WEDNESDAY, MARCH 11TH, 1891.

Association met at two o'clock, with President M. H. Fletcher in the Chair.

The Committee on membership presented the following names:

Dr. S. M. Cummings, of Elkhart, Ind.; Dr. J. F. Buxbaum, of Cincinnati; Dr. W. T. McLean, of Cincinnati.

On motion, they were elected to membership, by suspension of the rules and cast of the Secretary's ballot.

Dr. B. M. Ricketts, of Cincinnati, read a paper, "Surgery of the Cleft Palate."

Discussion was opened by Dr. Wm. Knight, followed by Drs. J. J. R. Patrick, E. S. Talbot, H. A. Smith, J. Taft and G. Mollyneaux, who presented for examination specimens illustrating the mechanical treatment of Cleft Palate.

On motion of Dr. H. A. Smith, the hearty thanks of the Association were tendered Dr. Ricketts for the excellent paper and the able manner of presenting the subject of "Surgery of the Cleft Palate."

Upon motion, the subject was passed and the discussion of Dr. Talbot's paper resumed by Drs. E. G. Betty, H. A. Smith and J. J. R. Patrick.

Upon motion, the subject was passed and Association adjourned, to meet Thursday morning.

SESSION, THURSDAY MORNING, MARCH 13TH, 1891.

Association met, with the President, Dr. Fletcher, in the Chair.

Minutes of the previous session were read and approved.

Bills to the amount of $———, were presented, approved and ordered paid.

Dr. J. I. Taylor, made the following motion: "That the Association endorse the amendment Dental Bill (House Bill, No. ——), now before the Ohio Legislature, and that a statement to this effect be telegraphed to Hon. Mr. McMaken, at Columbus, Ohio, passed.

On motion, 10:30 was the hour set apart for the election of officers.

The election of officers resulted as follows:
President, Dr. L. E. Custer, of Dayton, Ohio.
First Vice-President, Dr. O. N. Heise, of Cincinnati, Ohio.
Second Vice-President, Dr. Jessie Dillon, of Cincinnati, Ohio.
Corresponding Secretary, H. C. Matlack, of Covington, Ky.
Recording Secretary, Dr. H. T. Smith, of Cincinnati, Ohio.
Treasurer, Dr. F. A. Hunter, of Cincinnati, Ohio.

EXECUTIVE COMMITTEE.

Dr. J. R. Callahan, Cincinnati, Ohio.
Dr. O. N. Heise, Cincinnati, Ohio.
Dr. J. S. Cassidy, Covington, Ky.
Dr. Henry Barnes, Cleveland, Ohio.
Dr. L. P. Bethel, Kent, Ohio.
Dr. Wm. Conrad, St. Louis, Mo.
Dr. A. W. Harlan, Chicago, Ill.
Dr. B. O. Doyle, Louisville, Ky.
Dr. A. F. Emminger, Columbus, Ohio.
Dr. C. E. Miles, Columbus, Ohio.

PUBLICATION COMMITTEE.

Dr. L. P. Bethel, W. H. Sillito, Dr. F. W. Sage.

COMMITTEE ON MEMBERSHIP.

Drs. C. M. Wright, G. Mollyneaux, R. I. Taylor.

COMMITTEE ON ETHICS.

Drs. H. A. Smith, J. S. Cassidy, J. Taft.

H. T. Smith, Recording Secretary,

